# Hoffa’s Fat Pad Ganglion Cyst Causing Infrapatellar Impingement in a Collegiate Athlete: A Report of a Rare Case

**DOI:** 10.7759/cureus.103216

**Published:** 2026-02-08

**Authors:** Justin Prusinski, Angela Cavanna

**Affiliations:** 1 Primary Care, Touro College of Osteopathic Medicine, Middletown, USA

**Keywords:** hoffa’s fat pad, knee ganglion cyst, mri, musculoskeletal ultrasound, surgical excision

## Abstract

Ganglion cysts are benign, fluid-filled lesions most frequently arising from the wrist and hand, while occurrence within Hoffa’s (infrapatellar) fat pad of the knee is rare. When present in the knee, these cysts may lead to localized irritation, mechanical infrapatellar impingement, restricted range of motion, and anterior knee pain. We present the case of a 19-year-old collegiate basketball player with chronic anterior right knee pain without any prior history of trauma, unresponsive to initial conservative management. Initial clinical evaluation suggested patellofemoral pain syndrome; however, advanced imaging via MRI and musculoskeletal ultrasound revealed a ganglion cyst impinging on Hoffa’s fat pad and abutting the posterior patellar tendon. Given persistent functional impairment despite conservative therapy, arthroscopic excision was performed. Postoperatively, the patient achieved complete resolution of symptoms and successfully returned to competitive athletic activity without limitation. This case demonstrates the value of early imaging and consideration of intra-articular ganglion cysts in the differential diagnosis of anterior knee pain in young athletes. Early recognition through appropriate imaging is essential for optimizing functional outcomes in young athletes.

## Introduction

Ganglion cysts are benign, non-neoplastic cystic lesions enclosed by dense connective tissue containing gelatinous fluid, rich in hyaluronic acid and other mucopolysaccharides [1]. They most commonly occur around the wrist, hand, and foot. In rare cases, they can arise in the elbow or knee. Intra-articular ganglion cysts of the knee are uncommon, with those from the infrapatellar (Hoffa’s) fat pad being particularly rare, which often contributes to delayed or missed diagnosis [2]. MRI studies estimate the incidence of intra-articular ganglion cysts at 0.2-1%, with some reporting rates up to 1.3% [3]. This matches arthroscopic studies by Krudwig et al., who identified intra-articular cysts in about 1.1% of knees, most often incidentally [4].

The infrapatellar (Hoffa’s) fat pad is an intracapsular but extrasynovial structure. It can be affected by conditions ranging from benign to malignant processes, such as lipoma, hemangioma, osteochondroma, chondrosarcoma, ganglion cysts, and synovial chondromatosis [5]. Although often asymptomatic, intra-articular knee cysts can present with symptoms similar to chondromalacia, patellofemoral pain syndrome, meniscus injury, and chondral injury [6].

Although ultrasound is widely available and cost-effective, its diagnostic value may be limited for deep intra-articular lesions. MRI, however, is the gold standard because it provides superior soft-tissue contrast and higher diagnostic accuracy [7]. Symptomatic intra-articular ganglion cysts of the knee often require additional intervention, as conservative measures may be insufficient. Arthroscopic excision is the primary curative treatment [8].

We present a collegiate athlete with a symptomatic Hoffa’s fat pad ganglion cyst initially diagnosed as patellofemoral pain syndrome and managed conservatively. This example demonstrates the significance of early imaging and maintaining a broad differential diagnosis in young, athletic individuals, in whom delayed diagnosis may significantly affect performance and return-to-play timelines.

## Case presentation

A 19-year-old male collegiate basketball player presented with a two-month history of progressive anterior right knee pain. He denied any trauma or mechanical symptoms such as instability or locking. The pain was described as a dull ache localized to the infrapatellar region and exacerbated by knee extension, running, squatting, and jumping.

Patellofemoral pain syndrome was the initial diagnosis, based on activity-related anterior knee pain in the absence of structural abnormalities on examination. Conservative management, including non-steroidal anti-inflammatory drugs (NSAIDs), physical therapy, and activity modification, resulted in minimal improvement. Physical examination revealed mild tenderness over the infrapatellar region with limited terminal extension. Further stability testing revealed a negative anterior/posterior drawer, McMurray, and varus/valgus stress tests. There was also no evidence of joint effusion or neurovascular deficit.

**Figure 1:**
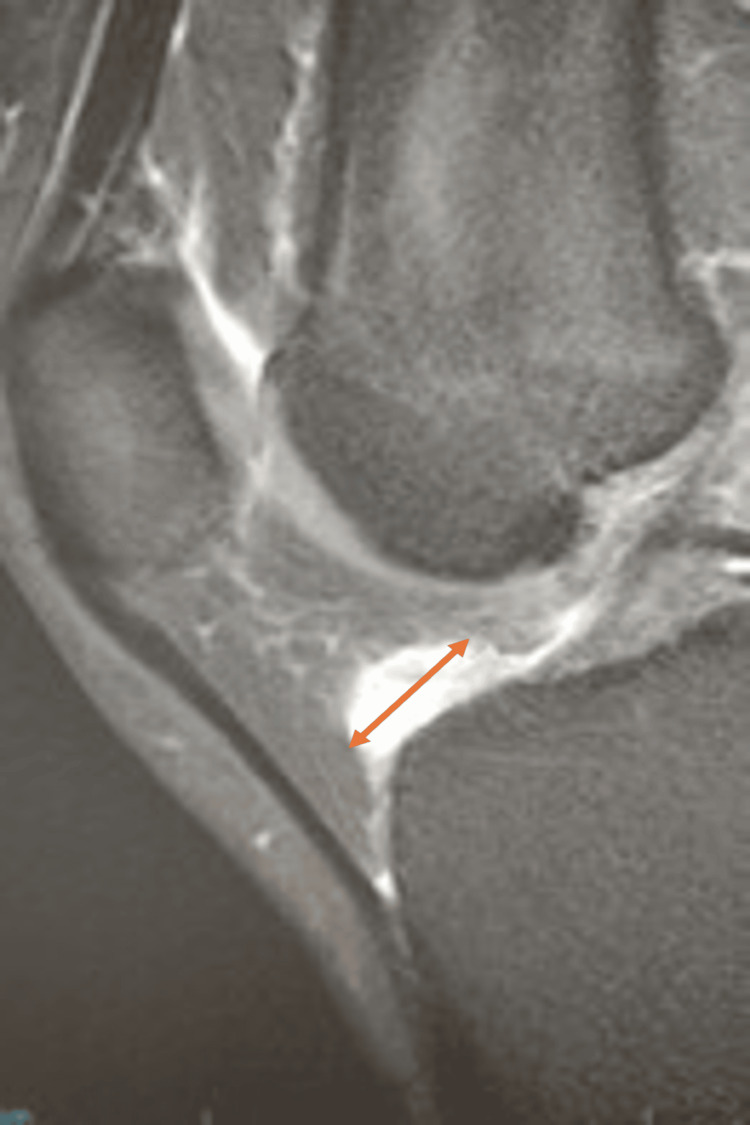
Sagittal T2-weighted MRI showing a well-circumscribed hyperintense ganglion cyst in Hoffa’s fat pad (arrow).

**Figure 2:**
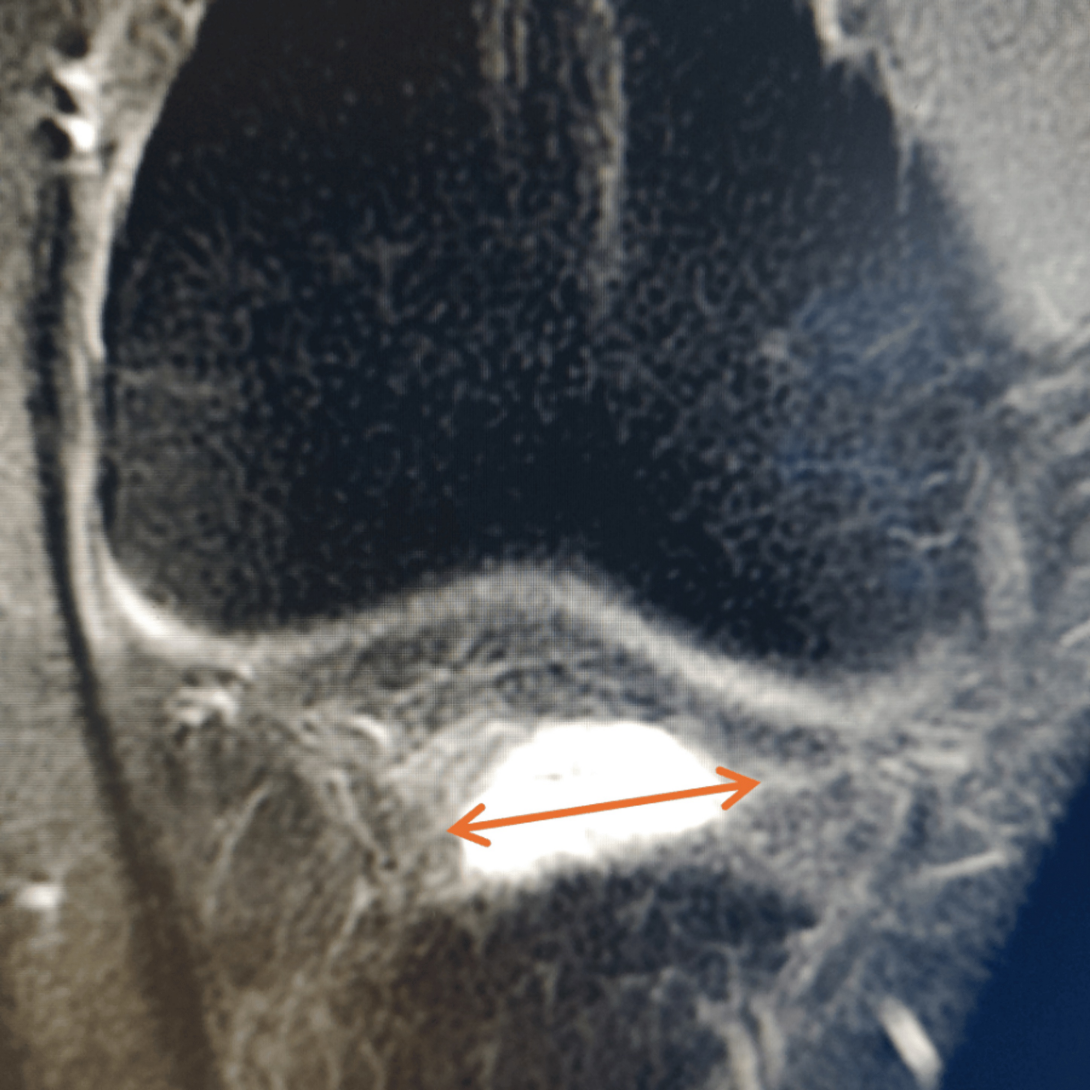
Coronal T2-weighted MRI demonstrating a ganglion cyst within Hoffa’s fat pad, anterior to the proximal tibia (arrow).

Given the patient’s refractory symptoms, an MRI of the right knee was obtained to accurately delineate the ganglion cyst and its stalk and to evaluate for any other lesions or associated intra-articular pathology that could influence management. Imaging revealed a well-defined, hyperintense cystic lesion between the anterior tibial plateau and Hoffa’s fat pad that was hyperintense on T2-weighted MRI, consistent with a ganglion cyst (Figure 1). Coronal MRI confirmed the lesion’s location anterior to the proximal tibia and inferior to the patella (Figure 2); no associated meniscal, ligamentous, or osseous abnormalities were identified.

Although nonsurgical options such as aspiration were considered, the cyst's deep intra-articular location and suspected stalk increased the risk of incomplete decompression and recurrence; therefore, the patient was referred to orthopedics for arthroscopic excision.

Postoperatively, the patient regained a full range of motion with complete resolution of his pain and has resumed full athletic activity without recurrence.

## Discussion

Hoffa’s fat pad ganglion cysts are rare lesions that account for approximately 0.2-1.3% of intra-articular knee cysts identified on MRI or during arthroscopy [3,4]. Their nonspecific clinical presentation often mimics other anterior knee pain, such as patellofemoral pain syndrome, leading to delayed diagnosis [6].

Hoffa’s fat pad ganglion cysts cause deep anterior knee pain at the patellar tendon region due to mechanical impingement between the patella, patellar tendon, and anterior tibial plateau. Pain worsens with knee extension, prolonged flexion, or dynamic movements like running, jumping, or squatting. Physical exams may reveal tenderness near the inferior patella and reduced motion, such as an extension block [9]. Provocative maneuvers, such as the Hoffa test, may reproduce the patient's symptoms during knee extension [9]. This case illustrates why intra-articular ganglion cysts should be considered when young athletes have persistent anterior knee pain unresponsive to therapy.

MRI remains the gold standard imaging modality for identifying intra-articular ganglion cysts due to its superior soft-tissue contrast and ability to localize lesions and assess their relationship to any adjacent structures [7]. Musculoskeletal ultrasound serves as a useful adjunct for confirming cystic characteristics and assessing any particular dynamic impingement, offering a more cost-effective and accessible option in clinical practice [7]. Nonetheless, early utilization of advanced imaging facilitates accurate diagnosis and timely intervention, ultimately minimizing any prolonged symptoms or functional impairment.

While conservative treatments such as physical therapy, NSAIDs, and ultrasound-guided aspiration are commonly employed as first-line interventions, these approaches can fail to provide adequate symptomatic relief when the cyst wall or stalk remains intact, predisposing patients to recurrence. Although aspiration is associated with a higher recurrence rate than surgical excision, it may provide temporary symptom relief and can be considered in select patients seeking minimally invasive management or who wish to avoid surgery [10]. In our case, arthroscopic excision was appropriate and successful because the cyst caused chronic mechanical impingement and was not amenable to conservative therapy.

Arthroscopic resection is regarded as the gold standard for symptomatic intra-articular ganglion cysts due to its minimally invasive nature and ability to achieve complete excision under direct visualization. However, cysts with extensive multiloculated architecture or extra-articular extension may require open excision to ensure complete removal. Gupta et al. reported cyst recurrence following incomplete arthroscopic excision, emphasizing the importance of complete resection to minimize recurrence risk [11]. In contrast, our patient’s cyst was localized and readily accessible, allowing for successful arthroscopic excision and complete symptomatic relief.

This case emphasizes the importance of maintaining a broad differential diagnosis when evaluating persistent anterior knee pain in active individuals. It also contributes to the limited literature describing intra-articular ganglion cysts arising from Hoffa’s fat pad, particularly in competitive athletes with delayed diagnosis. When conservative therapy fails or the clinical presentation remains unclear, advanced imaging with MRI and adjunctive ultrasound plays a critical role in identifying these lesions and guiding appropriate management decisions.

## Conclusions

Ganglion cysts from Hoffa’s fat pad are rare but important causes of anterior knee pain, especially in young, active individuals. Their symptoms often resemble common patellofemoral conditions, causing delays if advanced imaging is not used. MRI and ultrasound are key modalities for detection, anatomical characterization, and treatment planning. Conservative treatments may help briefly, but arthroscopic excision is best for persistent or mechanical symptoms. This case highlights the importance of early imaging and a broad diagnostic approach in assessing refractory anterior knee pain in athletes.
